# Establishment of an *in vitro* culture system to study the developmental biology of *Onchocerca volvulus* with implications for anti-*Onchocerca* drug discovery and screening

**DOI:** 10.1371/journal.pntd.0008513

**Published:** 2021-02-09

**Authors:** Narcisse V. T. Gandjui, Abdel J. Njouendou, Eric N. Gemeg, Fanny F. Fombad, Manuel Ritter, Chi A. Kien, Valerine C. Chunda, Jerome Fru, Mathias E. Esum, Marc P. Hübner, Peter A. Enyong, Achim Hoerauf, Samuel Wanji

**Affiliations:** 1 Research Foundation for Tropical Diseases and the Environment (REFOTDE), Buea, Cameroon; 2 Parasite and Vector Research Unit (PAVRU), Department of Microbiology and Parasitology, University of Buea, Buea, Cameroon; 3 Department of Biomedical Sciences, Faculty of Health Sciences, University of Buea, Buea, Cameroon; 4 Department of Zoology and Animal Physiology, Faculty of Science, University of Buea, Buea, Cameroon; 5 Institute for Medical Microbiology, Immunology and Parasitology (IMMIP), University Hospital Bonn (UKB), Bonn, Germany; 6 German Center for Infection Research (DZIF), Bonn-Cologne partner site, Bonn, Germany; National Institutes of Allergy and Infectious Diseases, NIH, UNITED STATES

## Abstract

**Background:**

Infections with *Onchocerca volvulus* nematodes remain a threat in Sub-Saharan Africa after three decades of ivermectin mass drug administration. Despite this effort, there is still an urgent need for understanding the parasite biology especially the mating behaviour and nodule formation as well as the development of more potent drugs that can clear the developmental (L3, L4, L5) and adult stages of the parasite and inhibit parasite reproduction and behaviour.

**Methodology/Principal findings:**

Prior to culture, freshly harvested *O*. *volvulus* L3 larvae from dissected *Simulium damnosum* flies were purified by centrifugation using a 30% Percoll solution to eliminate fly tissue debris and contaminants. Parasites were cultured in both cell-free and cell-based co-culture systems and monitored daily by microscopic visual inspection. Exhausted culture medium was replenished every 2–3 days. The cell-free culture system (DMEM supplemented with 10% NCS) supported the viability and motility of *O*. *volvulus* larvae for up to 84 days, while the co-culture system (DMEM supplemented with 10% FBS and seeded on LLC-MK_2_ feeder cells) extended worm survival for up to 315 days. Co-culture systems alone promoted two consecutive parasite moults (L3 to L4 and L4 to L5) with highest moulting rates (69.2±30%) observed in DMEM supplemented with 10% FBS and seeded on LLC-MK_2_ feeder cells, while no moult was observed in DMEM supplemented with 10% NCS and seeded on LEC feeder cells. In DMEM supplemented with 10% FBS and seeded on LLC-MK_2_ feeder cells, *O*. *volvulus* adult male worms attached to the vulva region of adult female worms and may have mated *in vitro*. Apparent early initiation of nodulogenesis was observed in both DMEM supplemented with 10% FBS and seeded on LLC-MK_2_ and DMEM supplemented with 10% NCS and seeded on LLC-MK_2_ systems.

**Conclusions/Significance:**

The present study describes an *in vitro* system in which *O*. *volvulus* L3 larvae can be maintained in culture leading to the development of adult stages. Thus, this *in vitro* system may provide a platform to investigate mating behaviour and early stage of nodulogenesis of *O*. *volvulus* adult worms that can be used as additional targets for macrofilaricidal drug screening.

## Author summary

River blindness affects people living in mostly remote and underserved rural communities in some of the poorest areas of the world. Although significant efforts have been achieved towards the reduction of disease morbidity, onchocerciasis still affects millions of people in Sub-Saharan Africa. The current control strategy is the annual mass administration of ivermectin which has accumulated several drawbacks over time, especially the action of the drug is solely microfilaricidal, very long treatment period (15–17 years) and reports of ivermectin losing its efficacy; thus, raising the urgent need for new adulticidal compounds. Our study has established an *in vitro* platform capable of supporting the growth and development of *Onchocerca volvulus* for up to 315 days, enabling the observation of parasite developmental processes: moulting (from the infective L3 stage to adults), increase in morphometry, the attachment of adult male and female worms and the potential initiation of nodulogenesis. Moreover, the platform might provide more insight into *O*. *volvulus* adult worms behavioural pattern *in vitro*. Also, our findings provide more avenues for mass production of different parasite stages, the investigation of parasite developmental biology and the identification of targets for drug discovery against different developmental stages of this filarial parasite within 315 days.

## Introduction

Little is known about the time course of *Onchocerca volvulus* parasite developmental processes, mating behaviour and nodule formation in the human host. Nevertheless, it is reported that moulting from L3 to L4 occurs within 3–7 days [1], while the L4 to L5 moult is estimated to occur after 2 months [2]. Early L5 are considered young adults and at this stage, the worms have partially developed gonads [3]. In the closely related *Onchocerca ochengi* cattle infection, it takes 279–532 days post-infection for the parasite to develop into fully mature and fertile adult worms capable of releasing microfilariae, the worms’ offspring [4]. More than 400 days post-infection is required for the same achievement in a chimpanzee model for *O*. *volvulus* [2,5].

Many non-conclusive attempts have been carried out to develop the complete life cycle of *O*. *volvulus in vitro* [1,3,6–16], though these contributions have been considered as important milestones towards achieving this ultimate goal. Serum/cell-free systems have been used in several studies to culture filarial parasites and reports have highlighted maintenance of fully viable parasites for a week [6–12]. Improvement of the culture conditions has been achieved by supplementing the basic culture media with serum or other culture ingredients (fatty acids and complex lipids formulation). The serum-based culture systems have been reported to support parasite longevity *in vitro* and cuticle casting [11,17–26]. Due to the inconsistency of serum composition, serum-free culture systems and co-culture systems using eukaryotic cells as feeder layers have been found successful [27–37,38–40]. Moreover, feeder cells were already shown to be crucial for *in vitro* cultivation and growth of *O*. *volvulus* [40,41]. From our previous observations on the *in vitro* growth and development of the filarial nematode *Loa loa* [36], among the three most used supplements (feeder layer, serum, and basic culture medium) for filarial parasite *in vitro* culture, feeder cells were classified as the most important requirement followed by the serum type and finally the nature of the basic culture medium. Summarily, the advancement of research towards the development of a suitable *in vitro* culture system for filarial parasites has highlighted the complexity of their requirement in terms of nutritional needs for growth and moulting from one stage to another.

This study aimed to identify the suitable *in vitro* culture requirements capable of supporting the maintenance and promoting the growth and development of the human parasite *O*. *volvulus* from its infective L3 larval stage to mature adult worms. Such an *in vitro* culture system will contribute to the experimental production of subsequent parasite stages (L4, L5 and adults), enable investigations on the behaviour of *O*. *volvulus*, as well as the probable initiation signs of nodule formation that could be used to further understand the parasite biology and identification of novel therapeutic drug targets against onchocerciasis.

## Methods

### Ethical statement

Ethical clearance was obtained from the National Institutional Review board, Yaoundé (N^0^ 2018/06/1057/CE/CNERSH/SP) after approval of the protocol. Before recruitment of the *O*. *volvulus*-infected individuals, the nature and objectives of the study were explained to potential participants and those who agreed to take part in the study signed a consent form. Special consideration was taken to minimize any health risks of the participant. They were followed-up for ivermectin treatment at the end of the study during the normal MDA period. Their participation was strictly voluntary and their documents were given a code for confidentiality.

### Determination of *O*. *volvulus* microfilarial load in skin biopsies of volunteers prior to *Simulium damnosum* engorgement

Participants examined were from the Meme drainage basin (overall Community Microfilarial Loads (CMFL) = 5.2 microfilariae/skin snip) and microfilarial load was determined as described by Wanji *et al*. [42]. Briefly, after the clinical examination, two skin biopsies from the posterior iliac crest were taken using a 2 mm corneo-scleral punch (CT 016 Everhards 2218–15 C, Germany). The skin samples from each participant were placed in two separate wells of a microtiter plate containing 2 drops of sterile normal saline. The corresponding well numbers were recorded in the participant’s form. The plates were sealed with parafilm to prevent any spillover or evaporation and incubated at room temperature for 24 hours. All emerged microfilariae were counted using an inverted microscope (Motic AE21) at 10x magnification and expressed per skin snip. Two participants were enrolled in the study that had average microfilariae load of 50 and 65 microfilariae/skin snip respectively.

### Collection of engorged *Simulium damnosum* flies

*Simulium damnosum* flies were collected along the banks of a fast-flowing river at Mile 16 Bolifamba (South West region–Cameroon). The fly collection team was composed of two trained individuals, one working from 07:00 am until 12 noon and the other from 12 noon until 6:00 pm for 5 consecutive days. Female blood-seeking *Simulium damnosum* flies were allowed to land on exposed legs of the microfilaridermic donor and blood-feed. After feeding, the flies were captured using *Simulium* rearing tubes and then transported to the laboratory insectarium. A maximum of 300 flies were allowed to feed on a volunteer every 48 hours. A preliminary entomological survey in the collection site showed very low parous and infection rates (0.05 and 0.02% respectively).

### Laboratory maintenance of engorged *S*. *damnosum*

Blood-fed *Simulium* flies were maintained in captivity under controlled experimental conditions as described by [43] for 10 days to allow ingested microfilariae to mature into infective stage larvae (L3). Captive flies were fed on 15% sucrose solution soaked in cotton wool and maintained at 23–28°C and 79–80% relative humidity.

### Dissection of flies, isolation and purification of *O*. *volvulus* third-stage larvae

After 10 days of rearing, a total of 1050 flies were dissected in Petri dishes (CytoOne, UK) containing RPMI 1640 medium (Sigma-Aldrich, St Louis, USA). The head, thorax and abdomen were separated and teased apart in three different dishes. Fly tissues were incubated for 20 min to allow L3 larvae to migrate out of the tissue. A sterile pipette was used to pick the larvae that were then pooled in a shallow convex glass dish [44]. Only L3 harvested from the head (where more mature larvae are expected to be found) were used in this study. The worms were transferred into 15 ml centrifuge tubes (Corning, Kennebunk-ME, USA) for purification using a Percoll (GE Healthcare, Pharmacia, Uppsala, Sweden) centrifugation technique as described by Zofou *et al*. [36]. In summary, the L3 suspension (340 L3), concentrated in less than 1 ml RPMI, was slowly layered on the surface of iso-osmotic Percoll and centrifuged (Humax 14k human, Germany) at 68 *x g* for 10 min. The process was repeated to remove microbial contaminants. In the end, the L3 were washed twice with RPMI-1640 by centrifugation at 239 *x g* for 10 min to remove Percoll remnant.

### Preparation of feeder cells and pre-conditioning in culture plates

Monkey kidney cells (LLC-MK_2_) are one of the most used feeder cells for the *in vitro* culture of filarial parasites, which have proven to boost parasite viability [12,32,35,36]. Thus, LLC-MK_2_ cells were selected for this study with three others (mouse lung embryonic cells (LEC), human embryonic kidney cells (HEK-293) and human hepatic cells (HC-04)) were selected for an exploratory purpose.

All the above-mentioned feeder cell lines were provided by the American Type Culture Collection (ATCC, Manassas, Virginia, USA). Feeder cells were cultured in flasks at 37°C in a humidified CO_2_ incubator (Sheldon Mfg. Inch, Cornelius, OR, USA) at 5% CO_2_ until the cell layer became fully confluent. For new inoculations and other cell manipulations, cells were dislodged with trypsin solution (25%) containing EDTA and kept at 37°C for less than 30 minutes. The cell suspension was centrifuged at 239 *x g* for 10 min, the supernatant discarded, and the pellet resuspended and diluted to 10^5^ cells/ml. Aliquots (100 μl) of cell suspensions were plated into each well of a 48-well flat-bottom culture plate and kept in the incubator until they became fully confluent and could be used for parasite maintenance in *in vitro* co-culture systems.

### *In vitro* culture of *O*. *volvulus* larvae

Harvested L3 from dissected flies were mixed and pooled to obtain parasite culture material. On average, the number of L3 per batch was 340 and this number varied among 5 batches (320–350 L3). Two sera supplements were used separately at 10% concentrations each: fetal bovine serum (FBS, Sigma- Aldrich, St Louis, USA) and new-born calf serum (NCS, Sigma-Aldrich, Berlin, Germany). Five basic media were used: RPMI-1640, IMDM, NCTC-135, MEM (Sigma-Aldrich, St Louis, USA), and DMEM (Gibco Life Technologies, Cergy-Pontoise, France). Penicillin-Streptomycin-Neomycin (PSN, 2%) was used as antibiotic and Amphotericin B (2.5 μg/ml) as an antifungal. Flat bottom culture plates (48-well) with lids (Corning, Kennebunk, ME, USA) were loaded as follows: For the co-culture systems, parasites (range 8–13 L3) in 1200 μl of the studied medium (basic culture medium + 10% serum) were loaded into a feeder-cell type pre-conditioned plate, while in cell-free systems they were loaded into empty wells. Five batches of infective L3 larvae were used throughout this study and each experimental culture system was carried out in quadruplet wells.

### Assessment of parasite viability

The viability of the parasites was assessed daily by visual inspection (by two individuals) under an inverted microscope until movement ceased. Their motility was scored on a 4-point scale as described in [45]. Briefly, score 0, no movement or immotile; score 1, intermittent shaking of head and tail; score 2, sluggish (shaking of the whole worm on a spot); score 3, vigorous movement (shaking of the whole worm and migration from one spot to another) was assessed. All experiments which supported viable parasites for up to 200 days were purposely stopped after 233 days, except for parasites that appeared to be mating. These parasites were monitored for up to 315 days (time at which the motility completely ceased).

### Parasite long term *in vitro* maintenance strategy

To achieve long term maintenance, 800 μl of exhausted culture medium was removed from each well and replaced with the same volume of fresh culture medium every 2–3 days. Additionally, cultured parasites were transferred from one culture plate (old) to another (new) either when feeder cells interfered with scoring parasite motility (parasites became entangled within overgrown cells) with the following cell lines (HC-04, LEC and HEK-293) every 2 weeks or when feeder cells (LLC-MK_2_) died every 7 weeks.

### Morphological features of mature *O*. *volvulus* male and female worms obtained *in vitro*

At the end of this study, stored (80% ethanol) female and male adult *O*. *volvulus* were processed for later viewing of the morphological features. Briefly, stored adult worms were rinsed with distilled water and cleared with Amann lactophenol (RAL diagnostics, Bordeaux, France) for 30 min. Before observations, cleared worms were flattened with a coverslip then viewed under a light microscope (HumaScope Plus, Wiesbaden, Germany) with 100X objectives and pictures were recorded using OPTIKA IS view software Version. 2.0 (Ponteranica, Ponteranica, Italy) giving a total magnification of 200X.

### Data processing and analysis

Three variables were used to assess the viability, growth and development of the parasites (mean motility, moulting rate and parasite stage morphometry). Raw data were saved in a spreadsheet and the percent motility was calculated according to the following formula:

$$Motility\;(\%)=\frac{\sum SiNi}{3.\sum Ni}\times 100$$

where Si is the score of point scale i and Ni is the total number of worms at a point scale i [45].

Filarial parasite moulting is one of the key phenomena providing clear evidence of worm growth and development. Moulted worms display casted cuticles and morphological changes. The moulting rate was calculated as previously described [35,36]:

$$Moulting\;rate\;(\%)=\frac{Number\;of\;casts\;counted\;(in\;well\;i)}{Total\;number\;of\;worms\;(in\;well\;i)}\times 100$$

(well i); is the culture well in which larval moulting rate is being evaluated.

Concerning parasite morphometry, photographs of *O*. *volvulus* at different stages were recorded with an inverted microscope and camera (OPTIKA, Ponteranica, Italy) and their length was determined using the OPTIKA IS view software Version. 2.0 (Ponteranica, Italy). ImageJ 1.52 software (NIH, USA) was used to generate scale bars of displayed photographs.

GraphPad Prism 8 software (GraphPad, San Diego, USA) was used to generate mean motility, moulting rate and morphometry graphs. Results of replicates were expressed as mean ± standard deviation (SD) for motility and moulting, while the median was used to summarise morphometric parameters of the parasite. The Kruskal-Wallis one-way analysis test was used to assess differences in motility, moulting rate and morphometry between sets of studied culture systems. Dunn’s *post-hoc* test was applied for pairwise multiple comparisons of the ranked data.

Factors that promoted parasite survival were identified using multiple linear regression. The general linear model (GLM) was built using the hierarchical stepwise method. A total of 4 blocks were achieved with the 5 factors (incubation time, presence or absence of feeder cells, basic medium, serum) and those that contributed significantly to the improvement of the model were identified based on the F-statistics and the adjusted R-square. The incubation time was treated as a metric factor. Dichotomous variables such as the presence of monkey kidney cells were coded using binary figures. For each nominal factor (Basic culture media, serum), sets of dummy variables were created and compared to one of the categories defined as reference. RPMI-1640 was used as a reference against DMEM, IMDM, MEM and NCTC. FBS serum was used as a reference against NCS.

The passage of *O*. *volvulus* larvae from the third (L3) to the fourth (L4) stages and L4 to the fifth (L5) stage was considered the second target product profile in assessing the suitability of the culture systems tested. Finally, the model was used to predict T_20_ and T_10_ values (Days), defined as the duration (incubation time) at which 20 and 10% of the worms, respectively, were still active (score 3). For all statistical comparisons, the *p*-values below 5% were evidence for rejecting null hypotheses.

## Results

Purified *O*. *volvulus* infective larvae were cultured in two distinct systems: the cell-free culture system and the cell-based co-culture system. The first step consisted of evaluating the potential of the cell-free systems (the combination of each of the five basic culture media and a single concentration of either of the two sera) on the viability, growth and development of *O*. *volvulus* larvae. The second step consisted of defining the best cell-free culture conditions for the four different mammalian cell lines to evaluate the beneficial effect of co-culture with feeder cell layers (co-culture systems).

### Evaluation of cell-free culture systems on the growth and development of *O*. *volvulus* larvae

**Fig 1:**
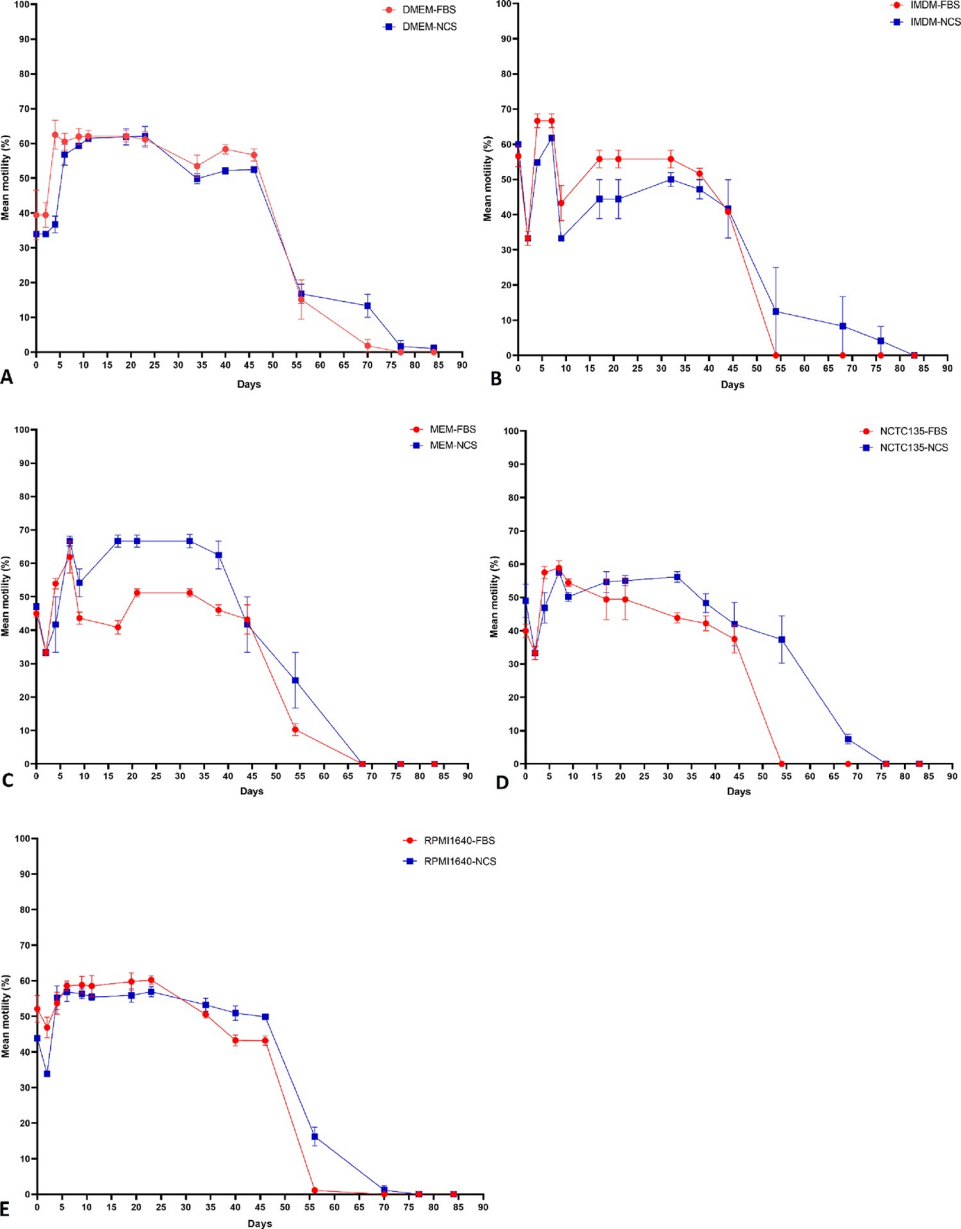
Motility pattern of *O*. *volvulus* (from L3 to L4) in the cell-free culture systems. **A** DMEM, 10% FBS/NCS. **B** IMDM, 10% FBS/NCS. **C** MEM, 10% FBS/NCS. **D** NCTC 135, 10% FBS/NCS. **E** RPMI1640, 10% FBS/NCS. (Results were pooled from different independent experiments [n = 3] and each experimental setting conducted in quadruplets). Dots represent mean motility and the ranges denote the standard error of the mean.

The cell-free system consisted of each of the five basic culture media supplemented with 10% of either NCS or FBS. The various study culture settings sustained *O*. *volvulus* larvae viability for a maximum of 84 days. Complete inactivity of all larvae was recorded in culture combinations IMDM, 10% FBS and NCTC135, 10% FBS after 54 days, and DMEM, 10% NCS and IMDM, 10% NCS after 84 days. Generally, freshly dissected and cultured *O*. *volvulus* infective L3 larvae were not vigorously active (motility score = 2, sluggish). Their motility significantly increased from day 3 to day 5 (motility score = 3, vigorously active) when the L3 stage larvae cast their cuticles to become L4. The parasite motility waned in all tested culture conditions after ~ 45 days of culture and was Medium-Serum combination dependent. Depending on the culture combination, complete parasite immobility was recorded between day 54 and day 84 (Fig 1).

**Fig 2:**
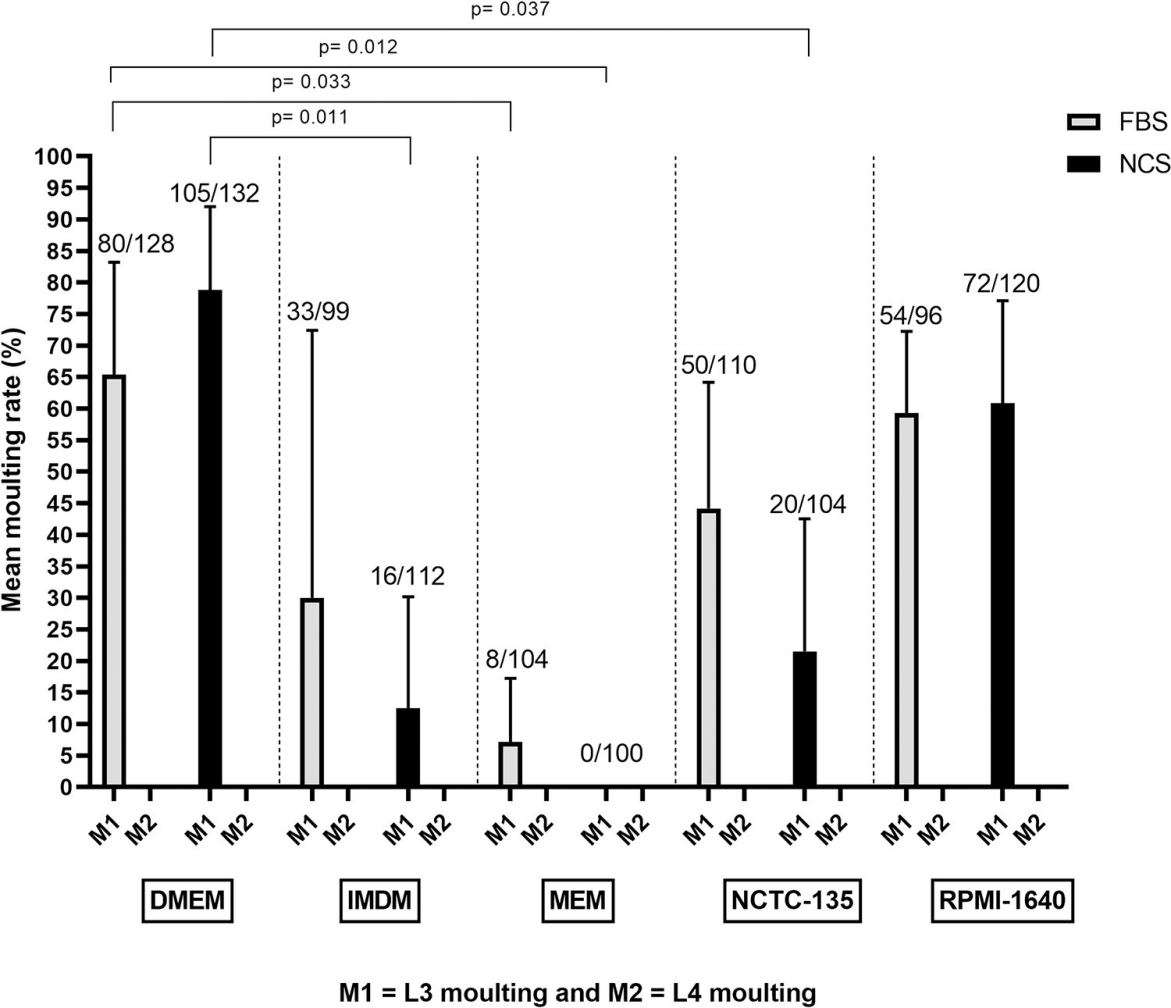
Influence of cell-free culture systems on *O*. *volvulus* larvae moults *in vitro*. Bars represent mean moulting rate, the ranges denote the standard deviation and fractions above bars stand for the total number of larvae moulted over the total number of larvae cultured. Results were pooled from different independent experiments [n = 3] and each experimental setting conducted in quadruplets.

*O*. *volvulus* moult was also used as an indicator to assess larval development *in vitro*. The moulting profile of *O*. *volvulus* larvae *in vitro* was culture system dependent and for cell-free systems, only the first parasite moult (L3 to L4 = M1) was observed. The moulting rate ranged from 0% (MEM, 10% NCS) to 78±13.2% (DMEM, 10% NCS). The culture system that best supported *O*. *volvulus* L3 moult was DMEM, 10% NCS, although no statistically significant difference was observed compared to all other combinations (Fig 2).

In cell-free systems, 3–5 days were required for *O*. *volvulus* infective larvae to moult from L3 to L4. Preliminary values of L3 and L4 length and width showed highly overlapping data with no significant differences, therefore focus was placed on comparing L4 and L5 values. All parasites that achieved the L3 to L4 moult failed to undergo the second moult (L4 to L5). In summary, the cell-free system supported parasite moult to L4 larvae and viability for up to 84 days but was below the timeframe necessary to allow adult parasite development.

### Evaluation of cell-based co-cultured systems on the growth and development of *O*. *volvulus* larvae

Since larvae did not become adults and their motility ceased by day 84 in the cell-free culture systems, we next evaluated the combination of the best cell-free system (DMEM-NCS/FBS) with each of the four mammalian cell lines (LLC-MK_2_, HC-04, HEK-293 and LEC) to improve parasite motility and viability as well as moulting.

**Fig 3:**
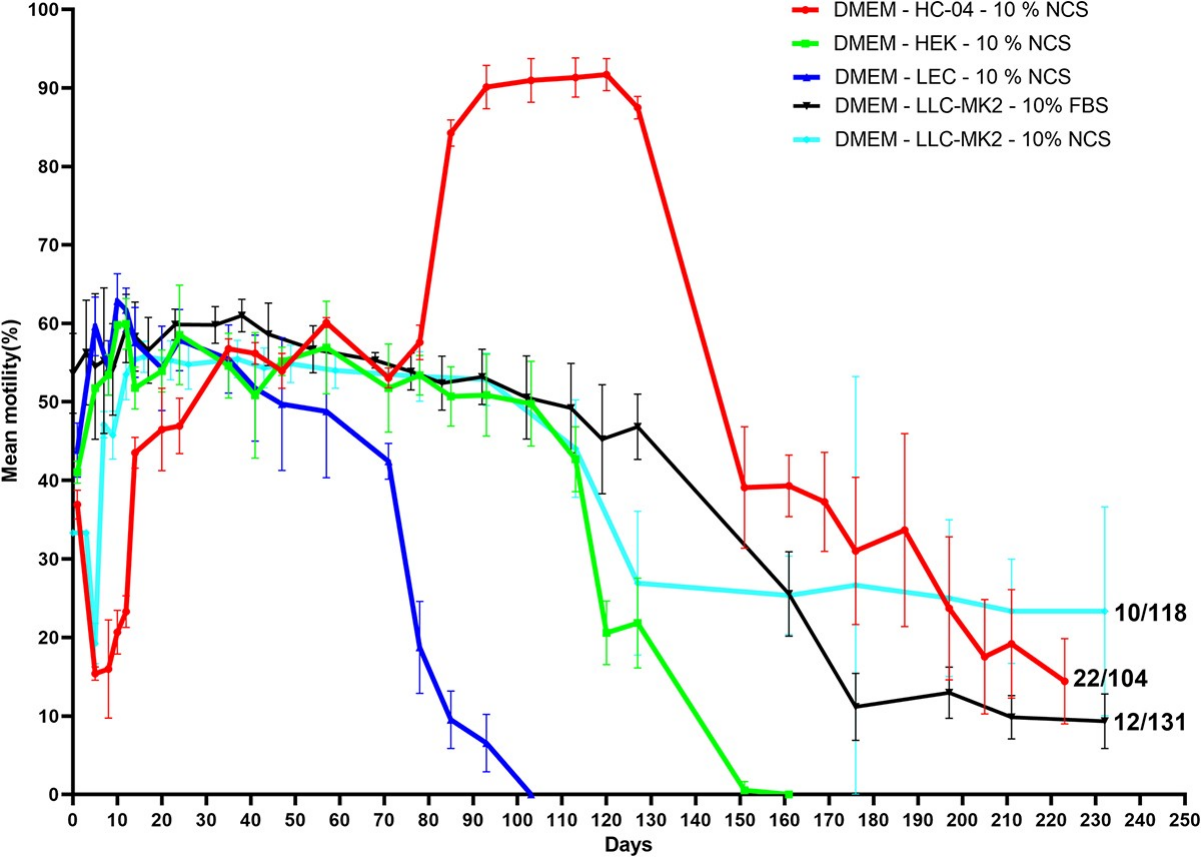
Motility pattern of *O*. *volvulus* larval stages in cell-based co-culture systems. Dots represent mean motility; ranges denote the standard error of the mean and figures highlighted on the curve represent the total number of active worms (Day 233) over the total number of worms cultured at the onset. Results were pooled from different independent experiments [n = 3] and each experimental setting conducted in quadruplets.

Interestingly, *O*. *volvulus* larvae survived for up to 315 days in cell-based co-culture systems although we purposely stopped all experiments that could support parasites viable for up to 200 days at day 233 (Fig 3). By day 233, 44/353 infective larvae developed into adult worms: 22/104 in DMEM, 10% NCS–HC04 cells, 12/131 in DMEM, 10% FBS–LLC-MK_2_ cells and 10/59 in DMEM, 10% NCS–LLC-MK_2_ cells (Table 1). Exception for prolonging parasite observation was given to 11 adult worms (11/353) that appeared to be mating. These parasites were monitored until day 315 (time at which their motility completely ceased). Thus, the culture of *O*. *volvulus* larvae on feeder cells increased their longevity by 3.75-fold as compared to those cultured in cell-free systems. As similarly observed in cell-free systems, cultured *O*. *volvulus* infective L3 larvae from freshly dissected *S*. *damnosum* flies did not display vigorous activity at day 0 *in vitro* (motility score = 2, sluggish). The infective larval (L3) motility significantly increased between days 3 and 7 (motility score = 3, vigorously active) when L3 stages cast their cuticles to become L4 larvae. The L4 larval motility remained rectilinear until day 48 when the first L4 to L5 larvae moult was observed. Except for larvae cultured in DMEM, 10% NCS-HC-04 cells, after day 48 L5 larval motility started dropping and movement completely ceased after 103 days in larvae cultured in DMEM, 10% NCS-LEC cells and after 162 days in larvae cultured in DMEM, 10% NCS-HEK cells. Increase in the motility of the L5 larval stage was solely observed in the culture system using DMEM, 10% NCS-HC-04 cells, with more than 90% of the parasites being very active until day 125. The motility of this group started dropping afterwards. At day 233, larvae cultured in DMEM, 10% NCS-HC-04 cells; DMEM, 10% NCS-LLC-MK_2_ cells; and DMEM, 10% FBS-LLC-MK_2_ cells still had motility of 10–20% (Fig 3).

**Table 1:** *O*. *volvulus* attrition disparity in different culture systems.

	Duration of *O*. *volvulus* in vitro culture						
Culture systems	Day 0	Day 54	Day 84	Day 104	Day 162	Day 233	Day 315
DMEM, 10% NCS-LLCMK2	118	118	117	94	30	10	6
DMEM, 10% FBS-LLCMK2	131	131	131	130	96	12	5
DMEM, 10% NCS-HC04	104	104	103	103	61	22	
DMEM, 10% NCS-HEK293	148	148	146	142	0		
DMEM, 10% NCS-LEC	105	105	28	0			
DMEM, 10% FBS	128	32	0				
DMEM, 10% NCS	132	36	0				
RPMI1640, 10% FBS	96	2	0				
RPMI1640, 10% NCS	120	30	0				
IMDM, 10% FBS	99	0	0				
IMDM, 10% NCS	112	24	0				
MEM, 10% FBS	104	16	0				
MEM, 10% NCS	100	34	0				
NCTC-135, 10% FBS	110	0	0				
NCTC-135, 10% NCS	104	38	0				

DMEM, Dulbecco’s Modified Eagle Medium; RPMI, Roswell Park Memorial Institute; IMDM, Iscove’s Modified Dulbecco Medium; MEM, Minimum Essential Medium; NCTC, New jersey Cell Type Collection; NCS, New-born Calf Serum; FBS, Fetal Bovine Serum; HC-04, Human Hepatocyte cells; HEK-293, embryonic human kidneys cells; LLC-MK2, Monkey Kidney cells; LEC, Mouse embryonic lung cells.

Values represent the total number of motile worms at different time point.

**Fig 4:**
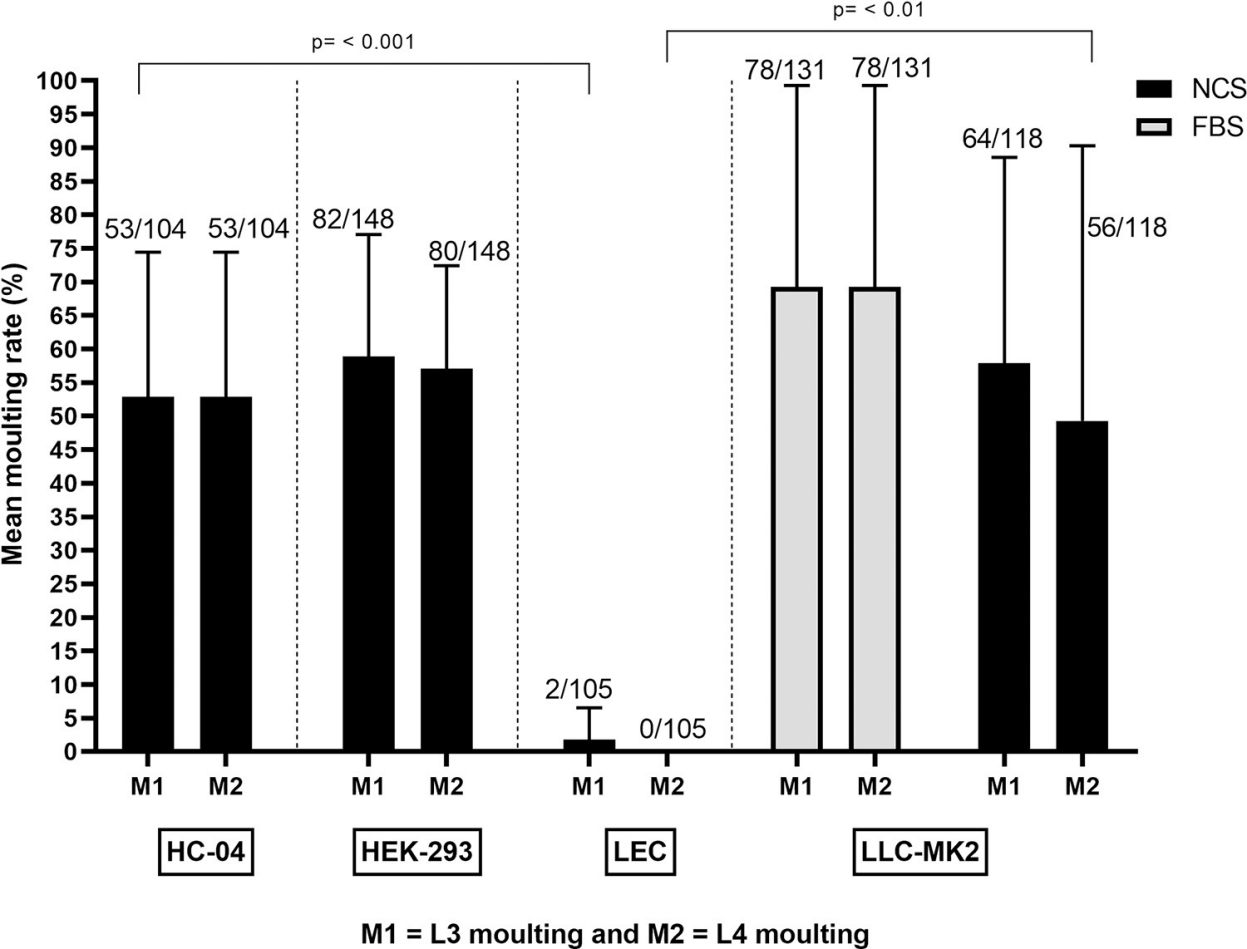
Influence of cell-based co-culture systems on *O*. *volvulus* larvae moults *in vitro*. Bars represent mean moulting rate, ranges denote the standard deviation and fractions above bars stand for the total number of larvae moulted over the total number of larvae cultured. Results were pooled from different independent experiments, n = 3 and each experimental setting conducted in quadruplets.

In contrast to cell-free systems, *O*. *volvulus* larvae underwent two consecutive moults in the cell-based co-culture systems. The first moult (L3 to L4 = M1) was observed within 3–7 days of culture onset while the second moult (L4 to L5 = M2) was observed between days 48 and 78, in which L4 larvae underwent cuticle ecdysis to become L5 larvae. The use of cell lines as feeder cell layer triggered the parasites’ second moult and it was consistently observed that amongst the larvae that achieved the first moult, the great proportion if not all underwent a successful second moult. *O*. *volvulus* moulting rates ranged from 0% (DMEM, 10% NCS–LEC cells) to 69.2±30% (DMEM, 10% FBS-LLC-MK_2_ cells). The cell-based co-culture system that best supported parasite moulting from L3 to L5 was DMEM, 10% FBS-LLC-MK_2_ cells (M1 = 69.2±30% and M2 = 69.2±30%), though no statistically significant difference was observed compared to DMEM, 10% NCS-LLC-MK_2_ cells (M1 = 57.8±30.7% and M2 = 49.2±41%); DMEM, 10% NCS-HC04 cells (M1 = 52.8±21.5% and M2 = 52.8±21.5%); and DMEM, 10% NCS-HEK293 cells (M1 = 58.8±18.2% and M2 = 57.0±15.3%). There was a statistical difference seen between larvae cultured with DMEM, 10% FBS-LLC-MK_2_ cells and larvae cultured with DMEM, 10% NCS–LEC cells (M1 = 1.7±4.7% and M2 = 0.0±0.0%) (Fig 4).

The length and width of *O*. *volvulus* larvae increased significantly throughout this study (233 days) and these changes were stage-dependent. *O*. *volvulus* larvae morphometry increased as larvae developed from one stage to another. At onset (Day 0), L3 larvae ranged from 590 μm to 626 μm in length and 16.7 μm to 23.4 μm in width. The L4 larvae varied from 1450 μm to 2122 μm in length and 44.7 μm–68.8 μm in width, while L5 larvae varied from 1478 μm to 3350 μm in length and 38.82 μm–114.5 μm in width. Although the length and width of both stages (L4 and L5) overlapped, male worms were shorter than female worms and a significant difference was observed between median length and width of L4 and L5 stages. The highest L5 larval lengths were observed in larvae co-cultured with LLC-MK_2_ cells in media supplemented with either NCS (length = 3327 μm, width = 81.5 μm) or FBS (length = 3350 μm, width = 114.5 μm).

### Divergence in morphometry pattern of *O*. *volvulus* male and female worms

**Fig 5:**
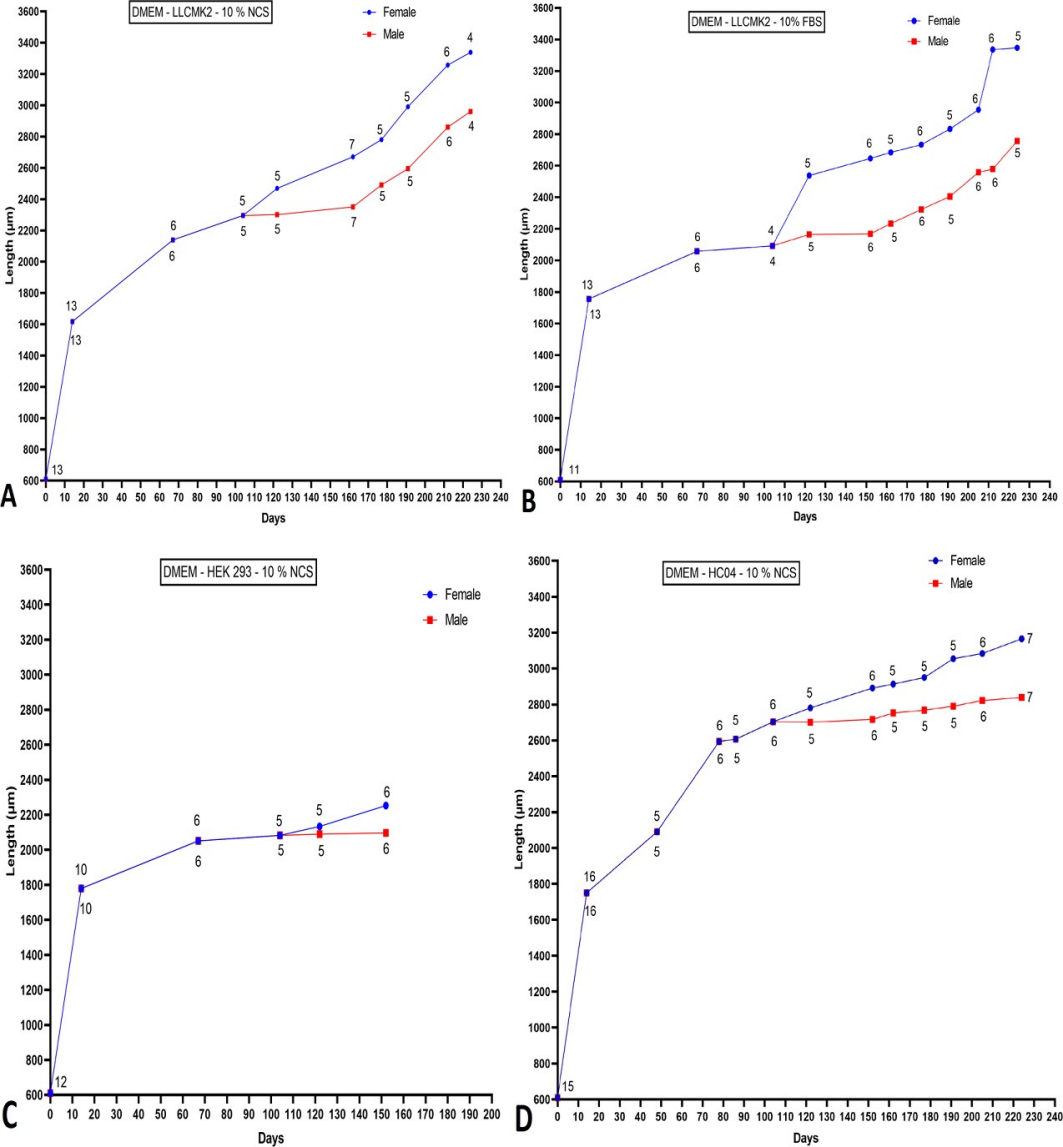
Disparity in sex-dependent worm morphometry changes in different cell-based co-culture systems. **A** DMEM, 10% NCS–LLC-MK_2_ cells. **B** DMEM, 10% FBS–LLC-MK_2_ cells. **C** DMEM, 10% NCS–HEK293 cells. **D** DMEM, 10% NCS–HC04 cells. Dots represent the median and figure above each dot denotes the number of worms measured at that given time point.

The length of female and male adult worms varied according to the cell-based co-culture system in which they were cultured. Among all cell-based co-culture systems used, only DMEM+10% NCS–LLC-MK_2_ cells; DMEM+10% FBS–LLC-MK_2_ cells; and DMEM+10% NCS–HC04 cells displayed the best appreciable differences. In general, no *O*. *volvulus* larvae sex differentiation was possible between days 0 and 104. Within this interval, the larval lengths had a wide variation but with no definitive conclusion as to worm sex was possible. After day 104, the wide variation in worm length values started dropping, leading to a better categorization of worms into sexes based on their length. *O*. *volvulus* adult male worms did not exceed 2900 μm while the female adult worms could reach up to 3300 μm (Fig 5).

### Evidence of *O*. *volvulus* adult worm maturity: adult male worm attachment to the vulva region of adult female worm *in vitro*

**Fig 6:**
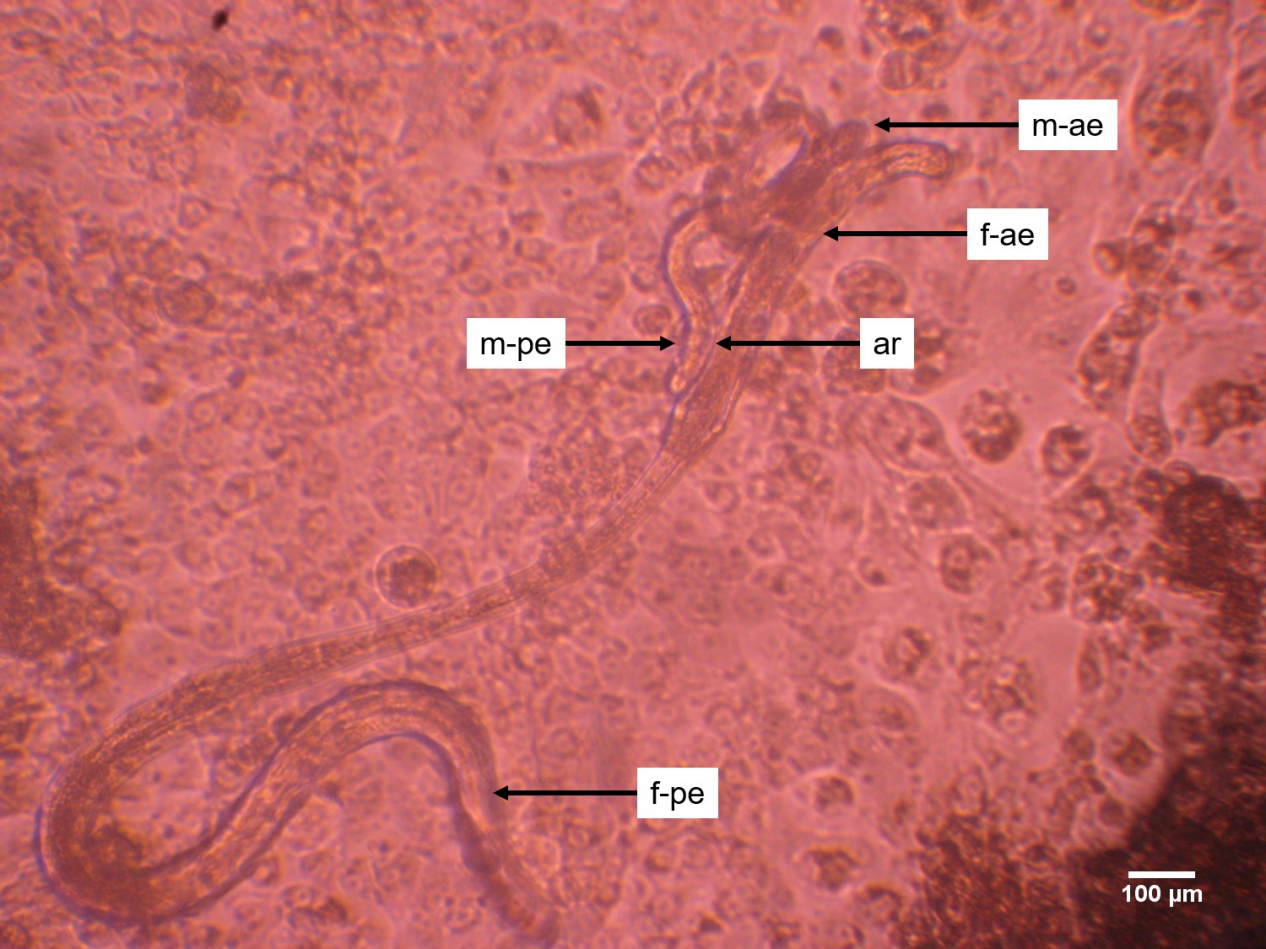
*O*. *volvulus* adult male and female worm entanglement *in vitro*. Adult male and female worm at day 212 involved in the attachment: The male worm posterior end (m-pe) is attached to the vulva region of the female worm (ar) which lasted for 12 days and could indicate possible mating. The male worm anterior end (m-ae) was coiled and facing the same direction as the female worm anterior end (f-ae).

Generally, experiments involving co-culture systems were monitored for up to day 233 except for DMEM, 10% FBS-LLCMK_2_ cells and DMEM, 10% NCS-LLCMK_2_ cells. Both were the sole co-culture systems which supported the growth, development and prolonged male worm attachment to the vulva region. Parasites were monitored in both systems for up to 315 days. Early L4 moults into L5 larvae were observed from day 48 until day 78. Newly moulted L5 larvae required an additional 4 months and 2 weeks (134 days) *in vitro* maintenance before the first adult male worm attached to the vulva region of the female worm and which appeared to be mating. Adult male worm attachment to the vulva region of the female worm was observed only in the DMEM, 10% FBS-LLCMK_2_ cell-based co-culture system. By day 212 of *in vitro* culture, the first *O*. *volvulus* adult male worm attached to the vulva region of the female worm (Fig 6 and S1 Media) and 12 days later, two adult male worms attached to the vulva region of the same adult female worm (S2 Media). The attachment of two adult male worms to the same adult female worm lasted 11 days after which only one adult male worm remained attached to the adult female worm. The successful adult male worm was attached to the vulva region of the female worm until day 294.

**Fig 7:**
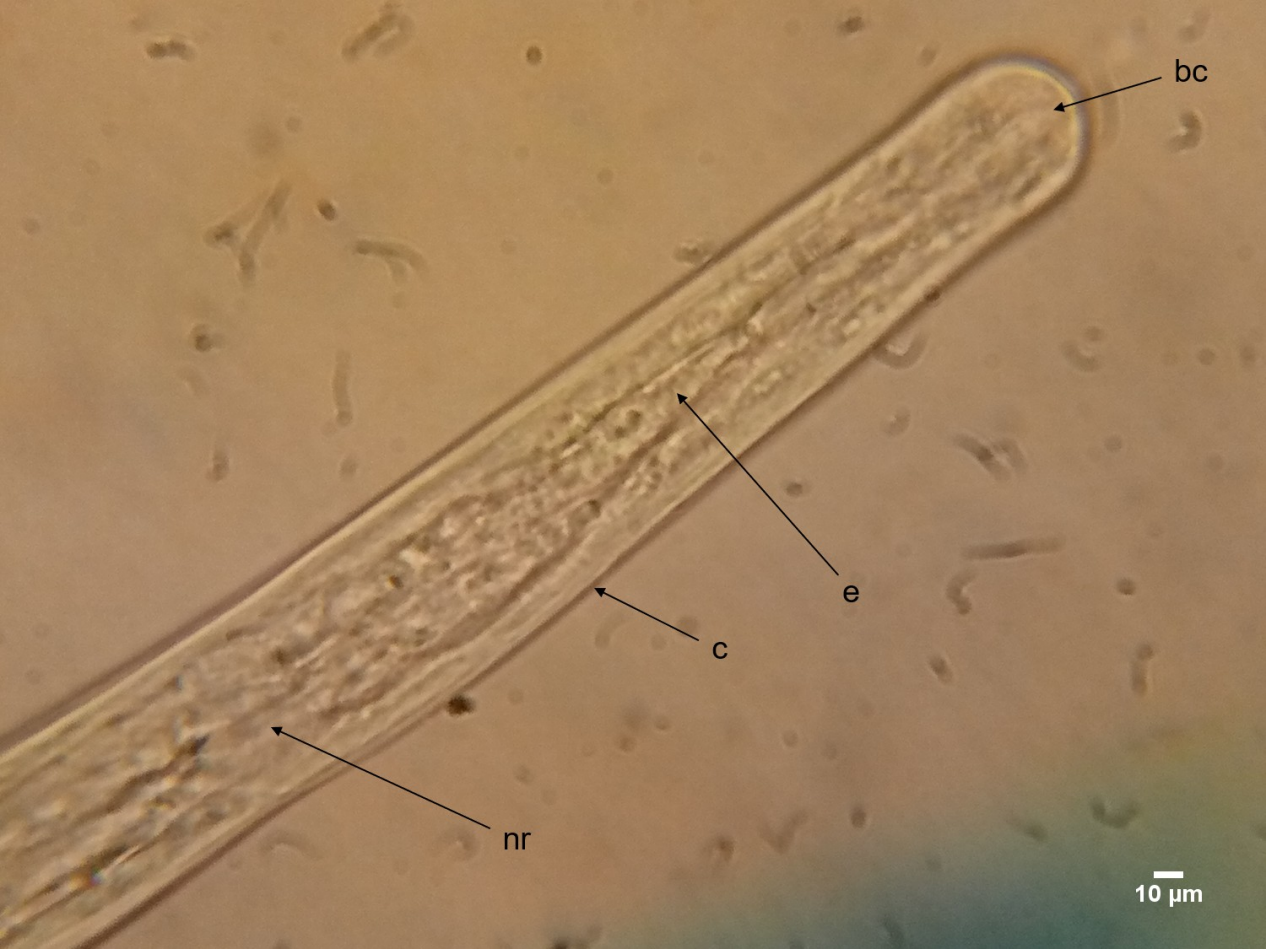
The anterior end of *O*. *volvulus* adult worm at day 233 (dorsoventral view). An overall view of the anterior end of adult *O*. *volvulus* worms highlighting the location of the cuticle (c), the buccal cavity (bc), oesophagus (e) and the nerve ring (nr).

**Fig 8:**
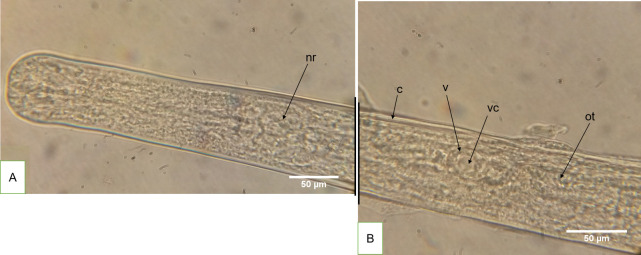
An adult female worm at approximately midbody at day 315 (ventral view). **A** Female adult worm anterior region by day 315 displaying well-developed nerve ring (nr). **B** Same female adult worm at approximately midbody showing the well-developed and opened vulva (v), which is a probable indication that mating had occurred *in vitro*, The cuticle is indicated by (c). The opened vulva exhibits a cavity (vc), which is connected to the ovijector tube (ot).

**Fig 9:**
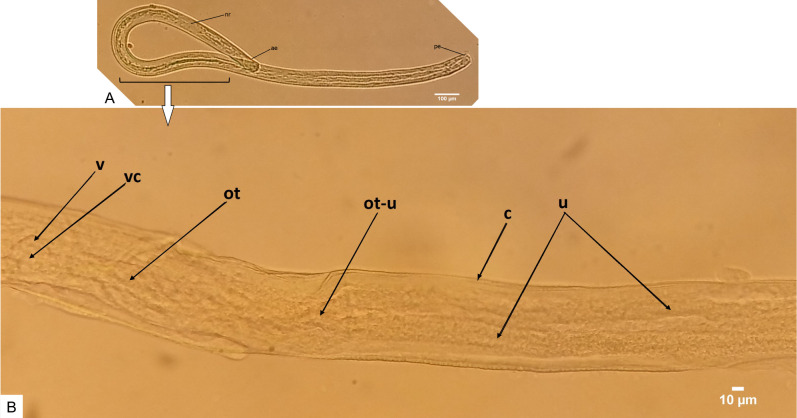
An adult female worm at day 315 displaying the inner reproductive organs (ventral view). **A** Overall morphology of adult female worm showing the anterior end (ae), the posterior end (pe) and the developed nerve ring (nr). **B** Adult female worm at approximately mid-body exhibiting an opened vulva (v) and the vulva cavity (vc) is directly connected to the ovijector tube (ot), which is posteriorly split into two separate tubes at the ovijector-uteri junction (ot-u) to form the uteri (u).

**Fig 10:**
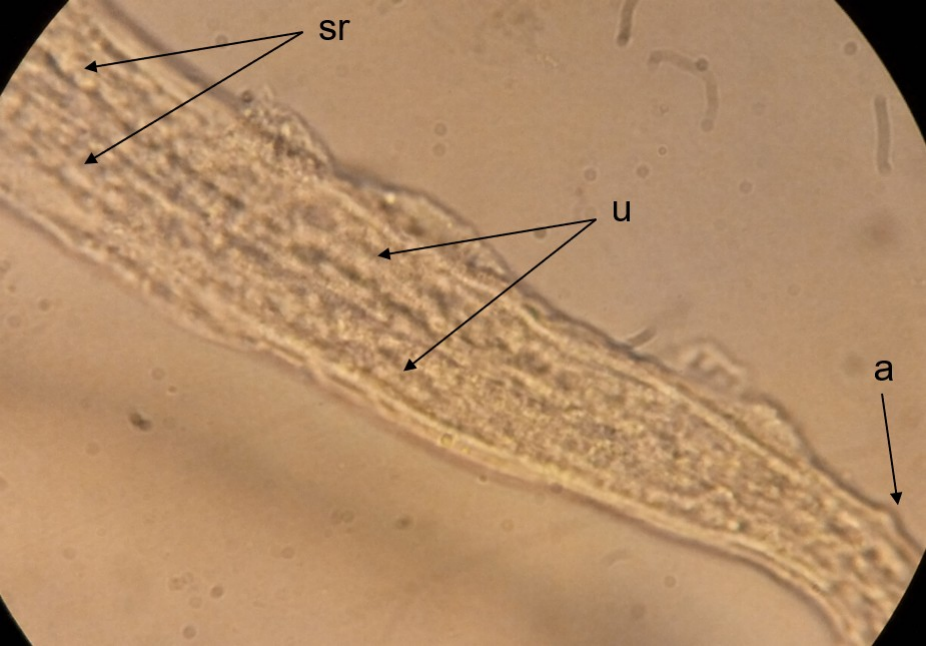
Posterior end of an adult female worm (lateral view). A female worm at day 315 with well-differentiated sigmoid-like seminal receptacle (sr) followed by the posterior end of the uteri (oviducts) and the anal opening (a).

**Fig 11:**
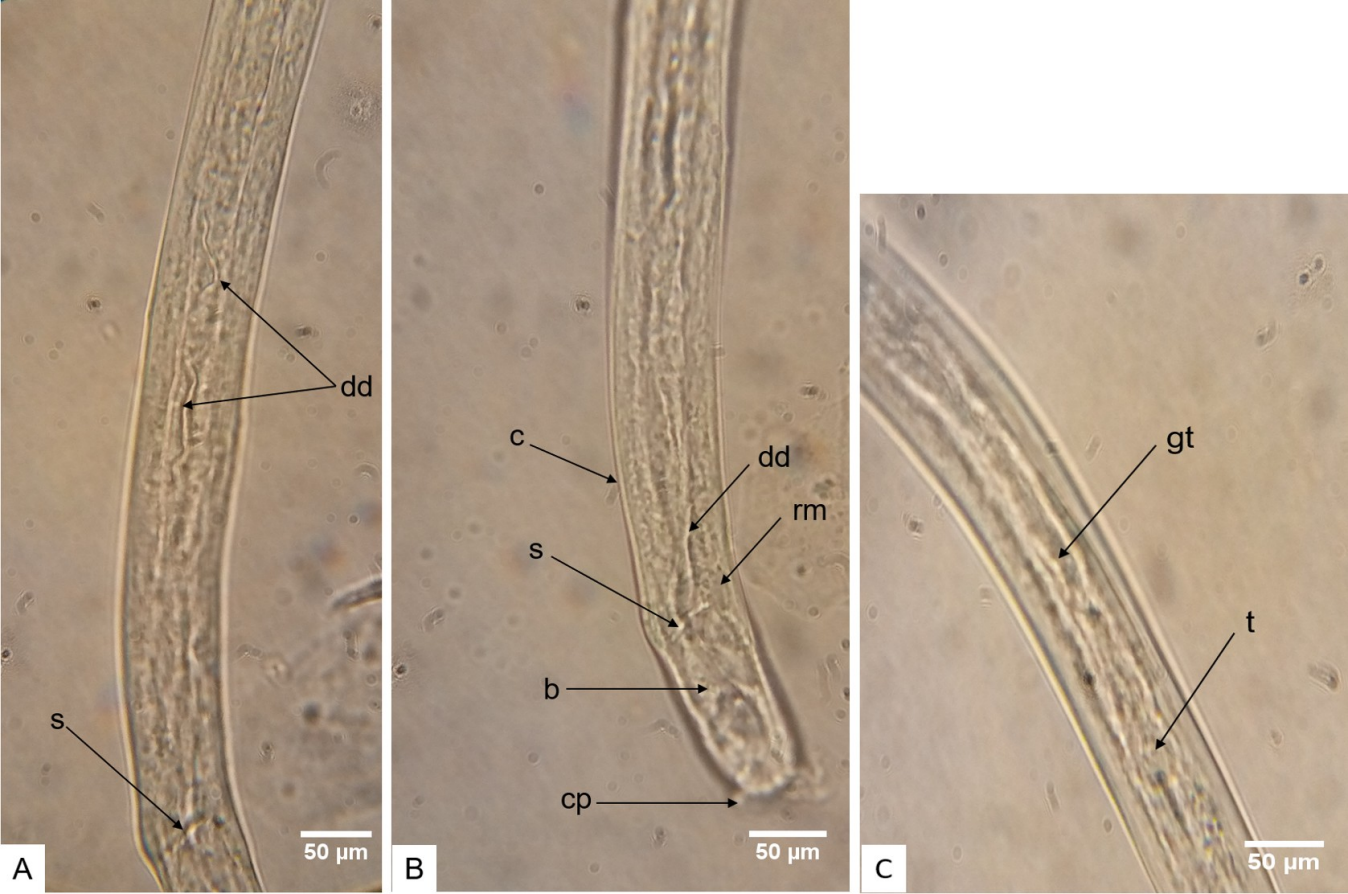
**Adult male worm posterior end exhibiting well developed genital features (A and B: lateral view) and developed testis approximately at male worm mid-body (C: lateral view). A and B** Male worm at day 315 displaying mature genital features, developed spicules (s) attached to retractor muscles (rm), the bursa (b), the ductus deferens (dd), the cuticle (c) and the caudal papillae (cp). **C** Mid-body of an adult male worm at day 233 illustrating the advance developmental state of the testis (t) C-shaped, curved posteriorly, with a high degree of cellular organization and the genital tube (gt).

The attachment process had a duration of 82 days and the parasites involved survived for another 3 weeks (S1 Fig)). The morphological features of these adult worms provide more evidence on their maturity (Figs 7–11) and probable mating *in vitro*. Adult female worms that experienced attachment on their vulva region with the adult male worms displayed a well-developed vulva and ovijector tube. Additionally, the vulva was wide opened suggesting that mating had occurred *in vitro* (Fig 8B). The uteri were also well-developed (Fig 9) leading to the sigmoid like shape of the seminal receptacles (Fig 10). Concerning the adult male worms, the morphological features showed well-differentiated spicules attached to retractor muscles which power spicules in and out of the male worm (Fig 11B). The development of adult male worm testes was advanced with a C-shaped, curved posteriorly, with a high degree of cellular organization (Fig 11C) and the tiny muscular tube in the male reproductive system that transport sperm to the ejaculatory ducts was clearly visible (Fig 11A).

Overall, the co-culture system (DMEM, 10% FBS-LLCMK_2_ cells) could sustain and support *O*. *volvulus* larvae for up to 315 days and resulted in parasite attachment. Out of 131 infective larvae (day 0) cultured in this system, 5 adult worms (3.81%) initiated prolong male-female worms’ attachment. Among the five worms were 2 female and 3 male worms.

### Presence of particulate matter around *O*. *volvulus* adult worms in the *in vitro* cell-based co-culture system

**Fig 12:**
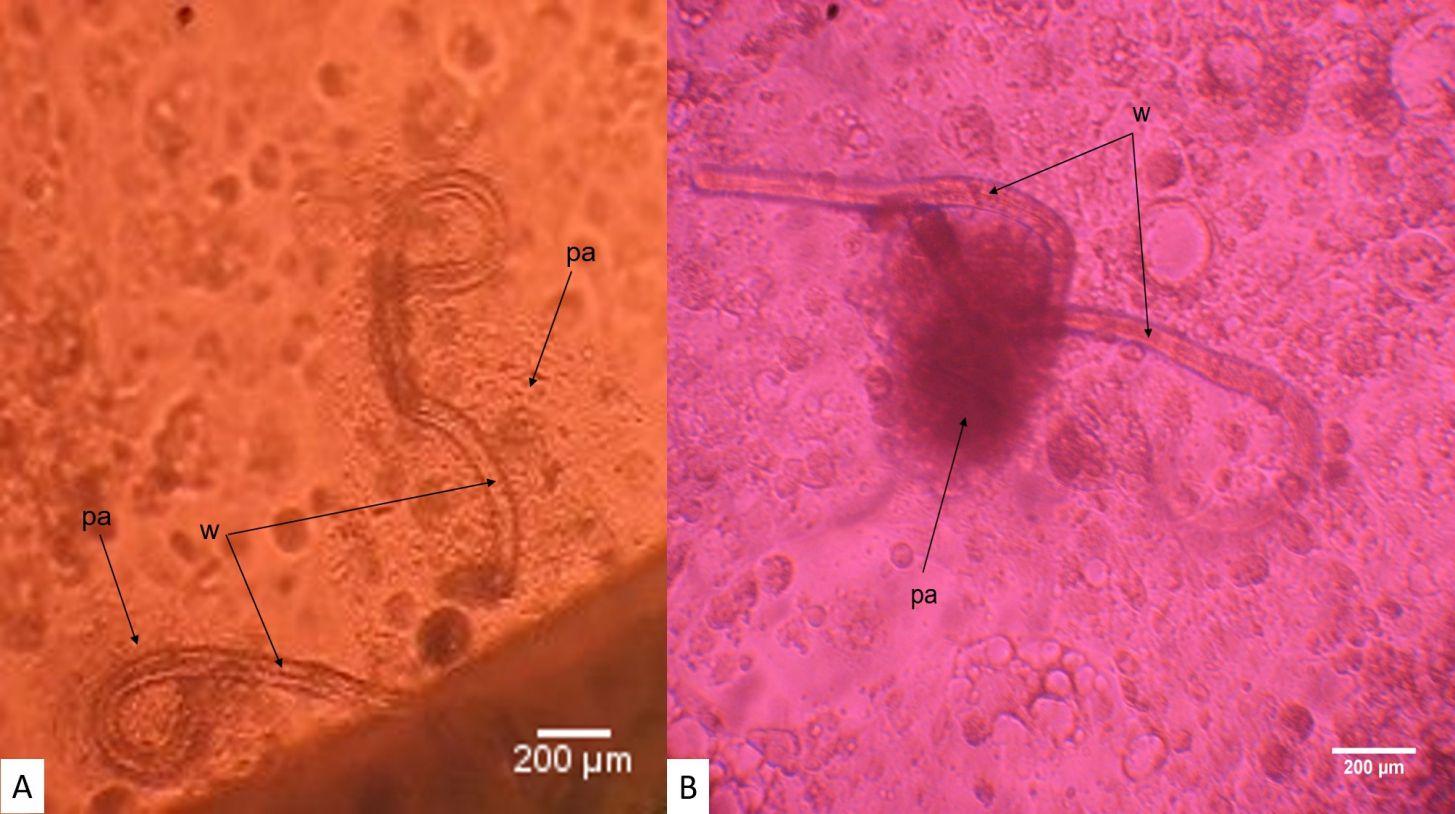
Early and late particulate matter adherence around *O*. *volvulus* young adults *in vitro* (day 177). **A** Early adherence of particulate matter (pa) along the worm (w) external wall. **B** Gathering of particulate matter (pa) into a globular/oval mass shape engulfing the young adult worms (w).

From day 177, we observed changes around some cultured worms notably particulate matter, which may suggest nodulogenesis is occurring around the *O*. *volvulus* L5. This feature was displayed only in co-culture systems, mainly with LLC-MK_2_ cells in combination either with DMEM, 10% NCS or DMEM, 10% FBS. The process started with the adherence of a thin and transparent particulate matter along the worm cuticle (Fig 12A). This transparent particulate matter later came together to form a globular or oval shape adhering around the worms that remained trapped in this mass (Fig 12B and S3 Media).

Particulate matter adherence lasted for 138 days after which parasites perished. Out of 129 *O*. *volvulus* infective larvae cultured in the cell-based co-culture system (DMEM, 10% FBS-LLCMK_2_ cells)_,_ 20.15% may have initiated early nodulogenesis while 16.9% initiated the process in DMEM, 10% NCS-LLCMK_2_ cells out of 59 infective larvae.

### Linear regression analysis: factors influencing the growth and development of *O*. *volvulus* larvae *in vitro*

The contribution of the various culture media and supplements used in the improvement of worm viability were identified based on their standardized coefficient (S1 Table). Among these factors, the presence of feeder cells pre-eminently influenced *O*. *volvulus* larvae viability, HC-04 feeder cells were classified as the topmost factor (β = 0.299) followed by LLC-MK_2_ feeder cells (β = 0.269), HEK-293 feeder cells (β = 0.129) and LEC feeder cells (β = 0.060). Although the basic culture media types also promoted *O*. *volvulus* larvae viability, their impact was less important than that of feeder cells but higher as compared to that of sera supplements. DMEM basic medium had the leading effect (β = 0.042) and the least effect was observed with IMDM (β = - 0.068 10^−3^). Both sera supplements, FBS (β = - 0.074) and NCS (β = - 0.052) had unfavourable effect compared to RPMI. The model was tested by assessing the assumptions of normal distribution and homoscedasticity. The histogram of the residuals (errors) in the model was used to check if they are normally distributed (S2 Fig). Although not perfect, the frequency distribution of the residuals displayed a shape close to that of the normal Gauss curve, indicating evidence of normal distribution. Additionally, the Q-Q plot was used for a further check (S3 Fig). Here, the theoretical and observed quantiles were closed, suggesting that the assumption of the normal distribution of the residual was far to be not violated. The model was used to predict T_20_ and T_10_ values (days) which correspond to the duration at which 20 and 10% of the worms were still active (score 3) (S4 Fig). Co-culture with feeder cells in DMEM medium represented the systems that could extend survival of parasites for a longer period.

## Discussion

This study was conducted to establish an *in vitro* platform that could support and promote the growth and development of *O*. *volvulus* larvae from the *S*. *damnosum*-derived L3 larvae to adult stages. Early studies that attempted to achieve this goal used serum/cell-free systems to culture filarial parasites and reported the maintenance with full viability for up to one week [6–12]. Our previous reports and those from other investigators highlighted the improvement of the culture conditions by supplementing the basic culture media used with serum or other synthetic additives. The serum-based culture systems achieved better parasite longevity and cuticle casting [11,14,17–24]. Due to challenges of the inconsistency of serum composition, other researchers opted to develop serum-free culture systems and co-culture systems using eukaryotic cells as feeder cell layers and yielded the best results obtained so far [16–27]. These studies did not provide a consensus on filarial parasite nutritional needs. However, Zofou *et al*. 2018 [36] established a hierarchical profile of most used *in vitro* culture supplements for filarial parasites in which the presence of feeder cells was ranked as most important followed by culture supplementation with serum and finally the culture media type. Based on these observations, four feeder layer cell line types (LLC-MK_2_, HC-04, HEK-293 and LEC), two serum types (FBS and NCS) and five basic culture media (RPMI-1640, DMEM, MEM, NCTC-135 and IMDM) were evaluated either in combination in the cell-free systems or in feeder layer co-culture systems for the growth and development of *O*. *volvulus* larvae *in vitro*.

To assess *O*. *volvulus* larvae *in vitro* growth and development, five variables were monitored: mean motility, moulting rate, parasite stage-specific morphometry, adult male and female worm attachment and the apparent initiation of nodule formation. Concerning *O*. *volvulus* larvae viability, parasite mean motility was used as the indicator. Regardless of the culture system, *O*. *volvulus* larval motility displayed an “upward and downward” pattern. For both systems (cell-free and co-culture systems), cultured L3 larval motility started reducing from day 0 and continued until day 3 when the first L3 moults were observed. The switch from one stage to another was in the first instance marked by a significant drop in motility followed by an abrupt increase in motility (shift from score 2 to score 3) and this same phenomenon was also later observed when larvae moulted from L4 to L5. The progressive drop in motility until moulting, followed by a drastic increase could be evidence that the greatest fraction of energy produced by the larvae is skewed towards cuticle ecdysis, which is prioritized at this time point with less energy assigned to worm movements. Moreover, it was observed that only co-culture systems could support *O*. *volvulus* larvae moulting from L4 to L5, further supporting that some nutrients secreted/excreted by feeder cells are key factors for parasite development. Toback *et al*. [46] reported that kidney epithelial cells release growth factors such as epidermal growth factor (EGF), transforming growth factor-alpha (TGF-α), insulin-like growth factor I (IGF-I), platelet-derived growth factor (PDGF) and insulin; which are exploited by the parasite for their growth and development. McConnell *et al*. [47] also showed that non-transfected HEK-293 cells release nerve growth factor that was beneficial to *O*. *volvulus* larvae growth and development. Without feeder cells, *O*. *volvulus* larvae stayed viable *in vitro* for up to 84 days, their longevity rose by 3.7-fold in the co-culture system (315 days). The co-culture system developed in this study made use of a single cell-based co-culture system (DMEM with 10% NCS or 10% FBS–LLC-MK2 cells) whereas Voronin et al. [41] used two distinct culture conditions to achieve the L5 stage. The maximum attainable longevity they reported for their system was 117 days versus 315 days for our new system. Additionally, our system achieved a higher L3 and L4 moulting rate (69.2±30%) as compared to theirs (Max. of 60%). Moreover, our single cell-based co-culture system supported adult *O*. *volvulus* worm attachment and triggered apparent early nodulogenesis. Malkmus *et al*, [40] evaluated the ability of engineered human skin and adipose tissue to facilitate the *in vitro* long-term development of *O*. *volvulus* and reported culturing L4 larval stage until they were 92 days old with a clear benefit of tissue-engineered skin models although these co-culture experiments do not yet represent methodologically sound culture systems for *O*. *volvulus*. Our findings open up new avenues for drug screening and in-depth investigation of *O*. *volvulus* biology.

Moulting entails synthesis of the new cuticle and shedding of the old one. Third-stage filarial larvae require two consecutive moults to become fully mature. The cell-free culture system could only support the first moult (M1) of *O*. *volvulus* infective larvae to L4 stage larvae ranging from 0% in MEM, 10% NCS to 78.8±13.2% in DMEM, 10% NCS. The second parasite moult (M2) was only observed in the co-culture system. It was therefore clearly established that the feeder cells play a crucial role in the development and maturation of *O*. *volvulus* parasite. Previous studies to culture other filarial parasites also demonstrated the essential role of feeder cells in their successful *in vitro* maintenance [20,35,36,41]. In the co-culture systems with DMEM, 10% NCS–HEK293 cells; DMEM, 10% NCS–LEC cells and DMEM, 10% NCS–LLC-MK_2_ cells, all *O*. *volvulus* L3 larvae that successfully undertook the M1 moults also achieved the M2 moults. The highest M1 and M2 moulting rates were reported in the co-culture system with DMEM, 10% FBS–LLC-MK_2_ cells (69.2±30.0% and 69.2±30.0%, respectively). This could be due to the fact that FBS has served as an additional source of essential nutrients, growth factors and hormones similar to monkey kidney cells. Moreover, FBS may bind and protect essential nutrients that otherwise are unstable. It could also function as a neutralizer of toxic substances in the medium and as a supplier of necessary transport factors or enzymes [48].

*O*. *volvulus* morphometry was also used as an indicator to assess parasite growth, although *O*. *volvulus* L4 stage morphometry overlapped with the L5 stages. The length of L4 stages ranged from 1450 μm to 2122 μm (Length median = 1794 μm) and width from 45 μm to 69 μm (Width median = 57 μm) and L5 length ranged from 1478 μm to 3350 μm (Length median = 1892 μm) and width from 39 μm–115 μm (Width median = 60 μm). There was no significant difference between L4 larvae measured from cell-free systems and L4 larvae from co-culture systems that failed to moult to L5 stages, whereas L4 larval lengths significantly differ from those of L5. The highest L5 larval length was recorded in the DMEM, 10% NCS–LLC-MK_2_ cells and DMEM, 10% FBS–LLC-MK_2_ cells co-culture systems. Moreover, L5 larval length from both systems differed significantly (*P =* 0.0418). We also noticed that outlier lengths recorded in co-culture systems using DMEM, 10% NCS–LLC-MK_2_ cells; DMEM, 10% FBS–LLC-MK_2_ cells and DMEM, 10% NCS–HC-04 cells corresponded to female *O*. *volvulus* larvae.

During this study, evidence of adult worm development was achieved. In filarial parasite biology, mating can only occur when parasites are mature with well-developed gonads. Moreover, these parasites have to be mature enough to be able to produce and respond to mating related signals for the process to be carried out. *O*. *volvulus* adult male worm attachment to the vulva region of adult female worms (82 days) was documented and photographed. The first prolonged male worm attachment to female worm vulva region was observed from day 212 as *O*. *volvulus* larvae were co-cultured in DMEM, 10% FBS–LLC-MK_2_ cells. Within 12 days, two adult male worms attempted to attach to a female. At the end of the process, only one male successfully attached to the female worm vulva region for a sustained time. The initiation of *O*. *volvulus* male worm attachment to female worms may have been triggered by attractants (hormones) excreted/secreted by the ready-to-mate female worm. This biological process involved five adult worms (2 females and 3 males) out of the 131 larvae cultured in this system and the process was initiated after 212 days of *in vitro* culture and lasted for 82 days, after which these parasites remained viable for 3 more weeks (21 days). The presence of chemotactic substances may have been responsible for this behavioural pattern involving 5 worms out of 131. Further investigations are needed to comprehensively describe this unique biological process and perhaps identify if the attachment biological process was triggered by chemotactic substances. The attachment biological process may have triggered the initiation of mating although we were limited with microscopy facilities for ultrastructure viewing. Further investigations on the sustained attachment of *O*. *volvulus* male and female worms using advanced imaging technologies will provide more information on adult worms mating process and the standard histology technique will confirm mating by the detection of spermatozoa in the developing female worm. Trees *et al*. 2000 reported that it takes 279–532 days post-infection (dpi) for the closely related *O*. *ochengi* parasite of cattle to develop into fully mature and fertile adult worms capable of releasing microfilariae and more than 400 days dpi for *O*. *volvulus* to do the same in a chimpanzee model [2,5]. Since the female worms survived only 3 weeks after attachment to male worms, we did not observe the release of microfilariae in this system. It’s possible that at this time point the female worms needed a specific stimulus either from the environment or self-produced to trigger embryogenesis and later release of microfilariae. This calls for further investigation to generate the complete reproductive cycle of *O*. *volvulus in vitro*.

*O*. *volvulus* adult worms which were involved in both the attachment and what appears to be the initiation of nodulogenesis survived for a longer period (315 days) as compared to those that failed to undergo these events (234 days). In the chronology of biological events, particulate matter was observed to adhere to *O*. *volvulus* adult worms from day 177, which was later exhibited as a linear adherence of these particulates along the parasites. The next event entailed male-female adult worm attachment that occurred beginning day 212 and finally, the particulate matter gathered into a globular/oval shape mass engulfing the affected parasites beginning on day 224. This event could indicate early nodulogenesis, however further studies are required to clarify the composition of the particulate matter as well as its role in worm development, e.g. an attempt for the parasite to produce a shelter to protect itself so that it can complete its developmental cycle as observed with nodule formation in the mammalian host. Further investigations might provide a clue on the origin of the material *Onchocerca* worms use to encapsulate themselves to form nodules since it has always been a topic of debates [49]. Electron microscopy of the probable early initiation of this biological process would have provided more insight into this aspect, but we were limited by the lack of this technology. According to Collins *et al*. [50], nodules only form around female worms and mating probably occurs before or early during nodule formation. The production of microfilariae by the female *O*. *volvulus* is not essential for nodule formation since many nodules contain non-fecund living females. These observations on the incomplete nodule formation *in vitro* deserves further investigations by providing to the *in vitro* system some immunologic effectors that exist *in vivo*.

The model was used to predict T_20_ and T_10_ values (days) which correspond to the time at which 20 and 10% respectively of the worms were still active (score 3). DMEM, 10% NCS–HC-04 cells had a very high motility trend compared to other cell-based co-culture systems. Our prediction model relied on worm motility and did not take into consideration other growth and development indicators (moulting and morphometry). Based on the parasite motility indicator only, the culture system DMEM, 10% NCS–HC-04 cells was classified as highest followed by DMEM, 10% FBS–LLCMK_2_ cells and DMEM, 10% NCS–LLCMK_2_ cells. Neither the attachment of adult male and female worms was observed nor the indicators for early nodulogenesis were recorded in DMEM, 10% NCS–HC-04 cells.

## Conclusions

This study has successfully established an *in vitro* platform for *O*. *volvulus* growth and development that mimics the parasite biology in the human host. The platform enabled us to culture *O*. *volvulus* for up to 315 days, supporting two consecutive parasite moults, displaying *O*. *volvulus* attachment behaviour, and the probable early initiation of nodule formation. The establishment of this platform therefore stands as an important achievement in *O*. *volvulus* developmental biology and has potential for the identification of targets for drug discovery against different stages of the development of this filarial parasite.

## Supporting information

S1 Media *O. volvulus* adult male and female parasites attachment *in vitro* at day 214 in DMEM, 10% FBS–LLC-MK2 cells culture system. (MP4)

S2 Media Two adult male *O. volvulus* parasites attachment to a female adult worm *in vitro* at day 228 in DMEM, 10% FBS–LLC-MK2 cells culture system. (MP4)

S3 Media Late adherence of particulate matter and engulfment of adult worms *in vitro* at day 205. (MP4)

S1 Fig Adult female *O*. *volvulus* worm (A) at day 233 (D) with clear cellular differentiation at the anterior region (D1) and posterior region (D2). Adult male *O*. *volvulus* worm at day 233 (E) with clear cellular differentiation of the anterior region (E1), with the well-developed digestive tract (E2).
(TIF)

S2 Fig Histogram of the residuals (errors) in the model for normal distribution and homoscedasticity. (PDF)

S3 Fig Q-Q plot suggesting normal distribution of the residual. (PDF)

S4 Fig Model predicted T_20 and_ T_10_ values in different in vitro culture systems (Days). (PDF)

S1 Table Experimental factors introduced in the model influencing *O. volvulus* larvae viability and their standardized coefficients. (PDF)
