# Supplementary figures and images for: Establishment of an in vitro culture system to study the developmental biology of Onchocerca volvulus with implications for anti-Onchocerca drug discovery and screening
Source: PLoS Negl Trop Dis. 2021 Feb 9;15(2):e0008513. doi: 10.1371/journal.pntd.0008513 (PMC7899360; doi:10.1371/journal.pntd.0008513)

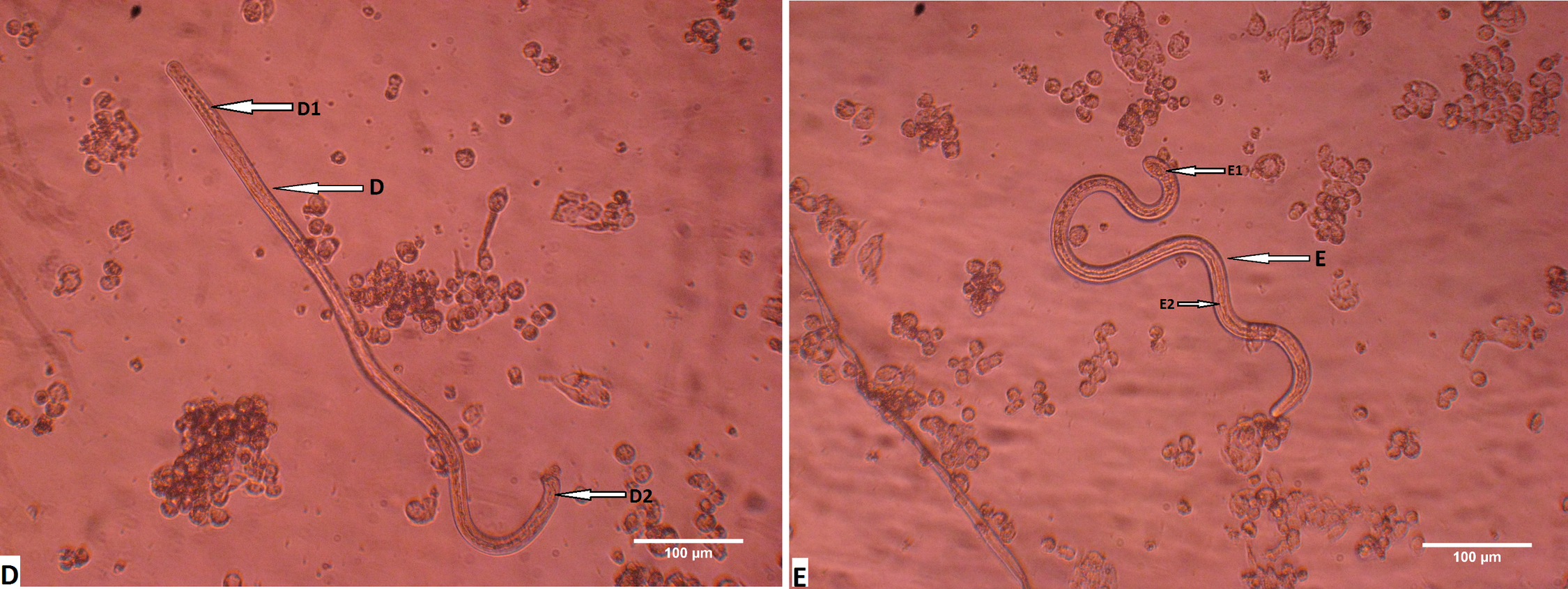

Supplement: S1 Fig — Adult female O. volvulus worm (A) at day 233 (D) with clear cellular differentiation at the anterior region (D1) and posterior region (D2). Adult male O. volvulus worm at day 233 (E) with clear cellular differentiation of the anterior region (E1), with the well-developed digestive tract (E2). (TIF) [file pntd.0008513.s004.tif]

## Histogram of Residuals

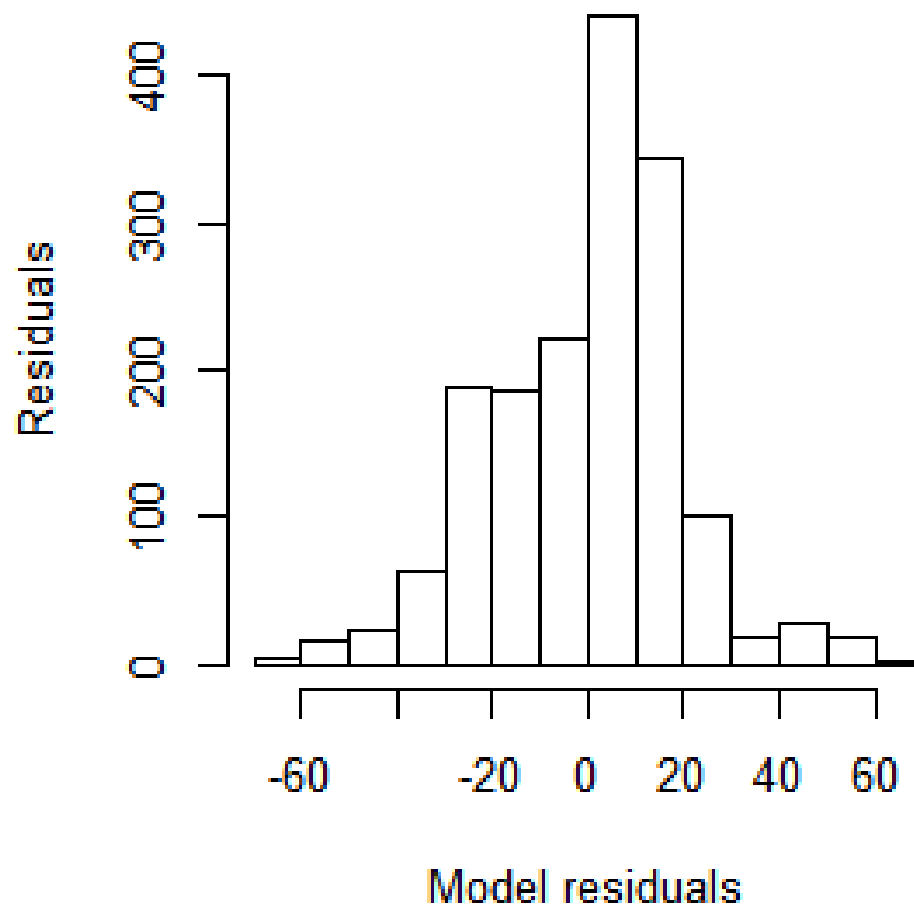

Supplement: S2 Fig — (PDF) [file pntd.0008513.s005.pdf]

Normal Q-Q Plot

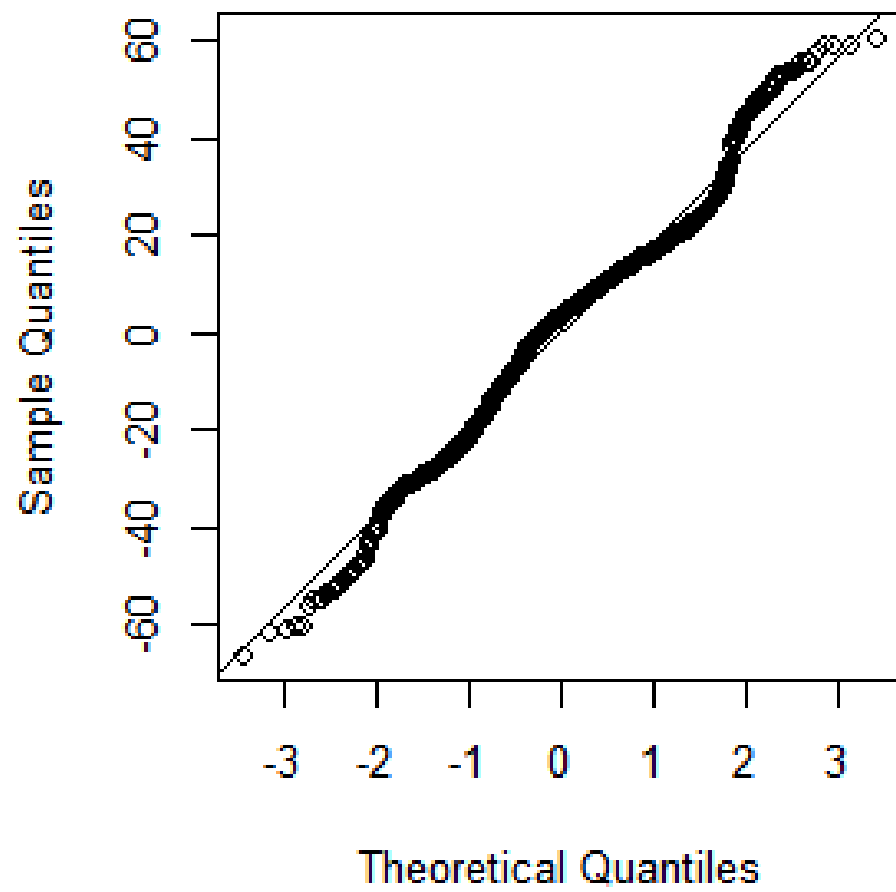

Supplement: S3 Fig — (PDF) [file pntd.0008513.s006.pdf]
