# Supplementary material for: Establishment of an in vitro culture system to study the developmental biology of Onchocerca volvulus with implications for anti-Onchocerca drug discovery and screening
Source: PLoS Negl Trop Dis. 2021 Feb 9;15(2):e0008513. doi: 10.1371/journal.pntd.0008513 (PMC7899360; doi:10.1371/journal.pntd.0008513)
Supplement: S4 Fig — (PDF) [file pntd.0008513.s007.pdf]

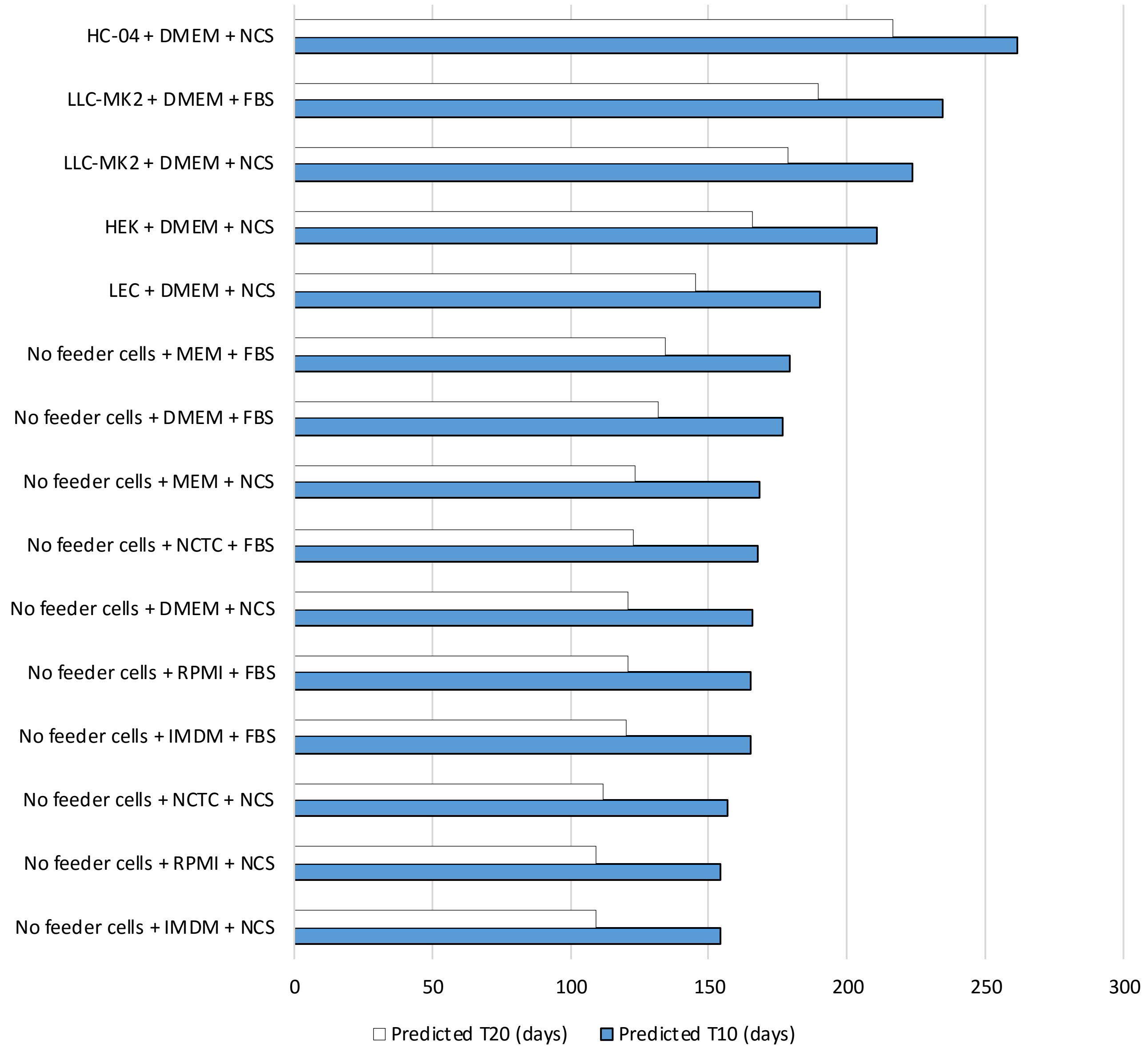

Feeder_layer	Medium	Protein	System tested	Predicted T10 (days)	Predicted T20 (days)
No feeder cells	IMDM	NCS	No feeder cells + IMDM + NCS	154.2018	109.2495
No feeder cells	RPMI	NCS	No feeder cells + RPMI + NCS	154.2351	109.2828
No feeder cells	NCTC	NCS	No feeder cells + NCTC + NCS	156.4671	111.5148
No feeder cells	IMDM	FBS	No feeder cells + IMDM + FBS	165.3439	120.3916
No feeder cells	RPMI	FBS	No feeder cells + RPMI + FBS	165.3772	120.4249
No feeder cells	DMEM	NCS	No feeder cells + DMEM + NCS	165.6388	120.6865
No feeder cells	NCTC	FBS	No feeder cells + NCTC + FBS	167.6092	122.6569
No feeder cells	MEM	NCS	No feeder cells + MEM + NCS	168.3225	123.3701
No feeder cells	DMEM	FBS	No feeder cells + DMEM + FBS	176.7809	131.8286
No feeder cells	MEM	FBS	No feeder cells + MEM + FBS	179.4645	134.5122
LEC	DMEM	NCS	LEC + DMEM + NCS	190.1696	145.2173
HEK	DMEM	NCS	HEK + DMEM + NCS	210.8403	165.888
LLC-MK2	DMEM	NCS	LLC-MK2 + DMEM + NCS	223.5371	178.5848
LLC-MK2	DMEM	FBS	LLC-MK2 + DMEM + FBS	234.6792	189.7269
HC-04	DMEM	NCS	HC-04 + DMEM + NCS	261.803	216.8507
