# Supplementary material for: Establishment of an in vitro culture system to study the developmental biology of Onchocerca volvulus with implications for anti-Onchocerca drug discovery and screening
Source: PLoS Negl Trop Dis. 2021 Feb 9;15(2):e0008513. doi: 10.1371/journal.pntd.0008513 (PMC7899360; doi:10.1371/journal.pntd.0008513)
Supplement: S1 Table — (PDF) [file pntd.0008513.s008.pdf]

Variables	Categories	Estimate (unstandardized coeeficient)	95% CI	Standardized coefficient (beta)	Std.Error	t-value	p-value
(Intercept)		46.79	[43.006 : 50.572]	0	1.9290	24.259	**2E-16**
Day		-0.2225	[-0.239 : -0.206]	-0.598	0.0082	-27.105	**2E-16**
Protein (Ref:FBS)	BCS	-5.239	[-8.502 : -1.977]	-0.07476	1.6630	-3.15	**0.00166**
	BSA-P	-5.284	[-14.256 : 3.689]	-0.02881	4.5740	-1.155	0.24825
	NCS	-2.479	[-5.091 : 0.133]	-0.0522	1.3320	-1.861	0.06288
Medium (Ref:RPMI)	DMEM	2.537	[-2.414 : 7.488]	0.04215	2.5240	1.005	0.31507
	IMDM	-0.007411	[-6.214 : 6.199]	-0.00006877	3.1640	-0.002	0.99813
	MEM	3.134	[-3.073 : 9.341]	0.02396	3.1640	0.99	0.32216
	NCTC	0.4965	[-5.71 : 6.703]	0.003796	3.1640	0.157	0.87534
Feeder cells (Ref: Cell free or No feeder cells)	HC-04	21.39	[16.681 : 26.104]	0.2992	2.4020	8.906	**2E-16**
	HEK	10.06	[5.26 : 14.851]	0.1297	2.4450	4.113	**0.000041**
	LEC	5.457	[0.362 : 10.552]	0.06051	2.5980	2.101	**0.03582**
	LLC-MK2	12.88	[8.888 : 16.872]	0.2694	2.0350	6.328	**3.2E-10**
